# Type I collagen aging impairs discoidin domain receptor 2-mediated tumor cell growth suppression

**DOI:** 10.18632/oncotarget.8795

**Published:** 2016-04-18

**Authors:** Charles Saby, Emilie Buache, Sylvie Brassart-Pasco, Hassan El Btaouri, Marie-Pierre Courageot, Laurence Van Gulick, Roselyne Garnotel, Pierre Jeannesson, Hamid Morjani

**Affiliations:** ^1^ Centre National de la Recherche Scientifique (CNRS), Unité Mixte de Recherche (UMR) Matrice Extracellulaire et Dynamique Cellulaire, Université de Reims Champagne-Ardenne, Unité de Formation et de Recherche (UFR) Pharmacie, Reims, France; ^2^ Centre National de la Recherche Scientifique (CNRS), Unité Mixte de Recherche (UMR) Matrice Extracellulaire et Dynamique Cellulaire, Université de Reims Champagne-Ardenne, Unité de Formation et de Recherche (UFR) Médecine, Reims, France; ^3^ Centre National de la Recherche Scientifique (CNRS), Unité Mixte de Recherche (UMR) Matrice Extracellulaire et Dynamique Cellulaire, Université de Reims Champagne-Ardenne, Unité de Formation et de Recherche (UFR) Sciences Exactes et Naturelles, Reims, France

**Keywords:** type I collagen, aging, cancer, discoidin domain receptor 2, cell proliferation, Gerotarget

## Abstract

Tumor cells are confronted to a type I collagen rich environment which regulates cell proliferation and invasion. Biological aging has been associated with structural changes of type I collagen. Here, we address the effect of collagen aging on cell proliferation in a three-dimensional context (3D).

We provide evidence for an inhibitory effect of adult collagen, but not of the old one, on proliferation of human fibrosarcoma HT-1080 cells. This effect involves both the activation of the tyrosine kinase Discoidin Domain Receptor 2 (DDR2) and the tyrosine phosphatase SHP-2. DDR2 and SHP-2 were less activated in old collagen. DDR2 inhibition decreased SHP-2 phosphorylation in adult collagen and increased cell proliferation to a level similar to that observed in old collagen.

In the presence of old collagen, a high level of JAK2 and ERK1/2 phosphorylation was observed while expression of the cell cycle negative regulator p21^CIP1^ was decreased. Inhibition of DDR2 kinase function also led to an increase in ERK1/2 phosphorylation and a decrease in p21^CIP1^ expression. Similar signaling profile was observed when DDR2 was inhibited in adult collagen. Altogether, these data suggest that biological collagen aging could increase tumor cell proliferation by reducingthe activation of the key matrix sensor DDR2.

## INTRODUCTION

Recent studies have emphasized the importance of bidirectional communication between neoplastic cells and their microenvironment in modulating tumor progression and metastasis, which represent the main uncontrolled problem in cancer therapeutics and the main cause of patient death [1]. The supporting players originating from surrounding normal tissue or from circulation include stromal fibroblasts, immune cells, vascular cells and the extracellular matrix (ECM) that can contribute both positive and negative signals to the tumor cells. Because of their pivotal role in tumor development and growth, the tumor microenvironment (TME) components are considered as challenging and attractive therapeutic targets.

During cancer initiation and progression, such a complex crosstalk involves dynamic interactions between tumor cells and ECM proteins, well-known to drive fundamental cellular functions including proliferation, differentiation, and motility. These interactions take place during the first steps of primary tumor growth, at the invasive edge for metastatic dissemination and during the initiation of secondary outgrowth in metastatic niches.

The main component of the interacting ECM proteins is fibrillar type I collagen that forms the mechanical backbone of interstitial tissues and constitutes up to 90% protein content [2]. Thus, this component is a privileged partner that can influence tumor cell proliferation. Type I collagen can be used as a pre-intravasation microenvironment and can be involved in the initiation of secondary outgrowth in metastatic niches.

Interestingly, due to its particular longevity with an estimated half-life of 15 years in humans, this key matrix protein appears as a preferential target for modifications during chronological and pathological aging, such as glycation that leads to Advanced Glycation End products formation (AGEs) [3]. These AGEs that interact with a specific receptor, AGEs receptor (RAGE), have been shown to play a role in tumor progression [4, 5]. Enzymatic modifications also induce an increase in collagen fibers cross-linking and consequently an alteration in type I collagen fibrillar properties [6]. Metalloproteinases have also been shown to alter type I collagen integrity [7]. These enzymatic modifications are able to impact tumor progression [8, 9].

At the cellular level, different transmembrane type I collagen receptors have been identified: integrins, the most studied receptors [10] and Discoidin Domain Receptors DDR1 and DDR2, the least explored ones [11]. In collagen molecules, the specific consensus sequence motifs GFOGER [12] are recognized by the heterodimeric integrins α1β1 and α2β1. DDR1 and DDR2 differ from the integrins in that they belong to the tyrosine kinase receptor (RTK) superfamily. DDR1 and DDR2 recognize GVMGFO motifs [13]. Unlike classical growth factor activated RTKs, such as the EGFR receptors which display rapid and transient activation [14], DDR1 and DDR2 are unique in that they exhibit delayed and sustained phosphorylation upon binding to collagen [15].

In the present study, we evaluated the influence of adult and aged three-dimensional (3D) collagen matrices on the proliferation of human HT-1080 fibrosarcoma cells. It is important to note that 3D collagen matrices are *in vitro* culture models closest to *in vivo* microenvironment. A significantly high cell proliferation rate was observed in old collagen compared to the adult one. This led us to investigate which actor among the receptors cited above, RAGE, integrins or DDRs, might be responsible for the effects observed. The present study demonstrates that DDR2 - as a key component of type I collagen-cell interaction and signaling - leads to differential regulation of cell proliferation between adult and old 3D collagen matrices.

## RESULTS

### Effect of aging on type I collagen properties

**Figure 1:**
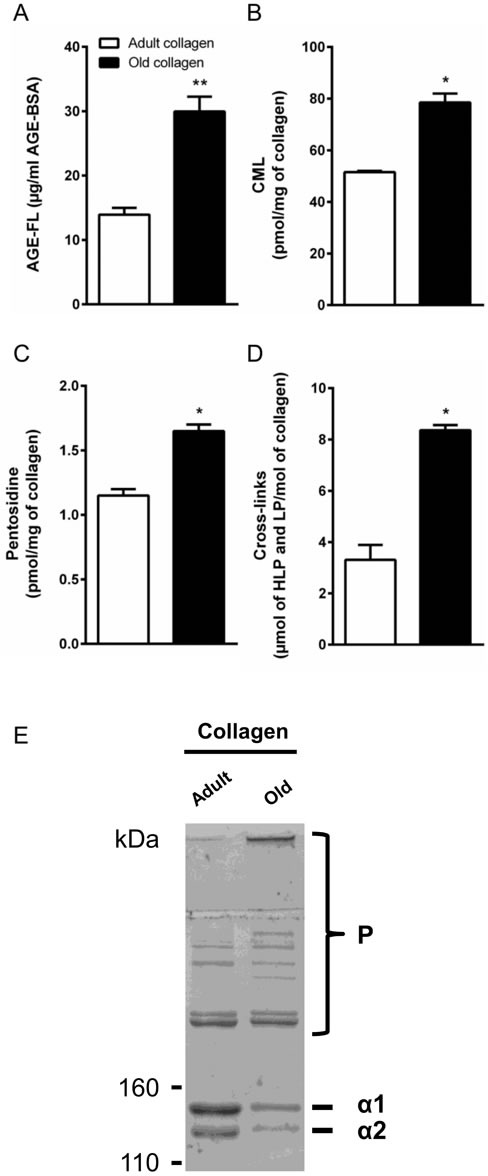
Characterization of collagens **A.** Spectrofluorimetric analysis was performed on adult and old collagen to detect AGEs-specific fluorescence expressed as μg/ml. **B.** CML and **C.** Pentosidine were quantified by LC-MS/MS and expressed as pmol/mg of collagen. **D.** Cross-link content was measured by the quantification of hydroxylysylpyridinoline (HLP) and lysylpyrodinoline (LP) by ion exchange chromatography and expressed as μmol (LHP and LP)/mol of collagen. **E.** SDS-PAGE of collagen samples, 5 μg of either adult or old rat type I collagens were analyzed on 5% polyacrylamide gels under reducing conditions. Collagen chains (α 1 and α 2), and higher-molecular-weight polymers (P) are indicated. Values represent the mean ± S.E.M. of three independent experiments (*p < 0.05, **p < 0.01).

Type I collagen was extracted from tail tendons of rats aged 2 months (adult) and 2 years (old) as described in the material and methods section. For each extraction experiment, ten animals were used for each age regardless of sex. Data previously obtained have shown that proliferation rate of HT-1080 cells was similar in collagen from males and females (data not shown). Then, collagens have been characterized according to the properties associated with the process of aging. First we analyzed advanced glycation endproduct (AGE) load which is commonly increased in aged-tissue, especially in long life proteins such as collagen [16, 17]. AGE content was assessed by detecting total AGEs quantified by fluorescence spectroscopy, and specific AGEs Nε-(Carboxymethyl) lysine (CML), and pentosidine by LC/MS/MS. As expected, age-dependent analyses showed that the level of fluorescing AGEs, CML and pentosidine, increased in collagen prepared from old rats compared to adult ones (Figure 1A-1C). Enzymatic cross-link content, known to be modified during aging [17], was then analyzed. As shown in Figure 1D, old collagen exhibits a higher concentration of the cross-links hydroxylysylpyridinoline and lysylpyrodinoline compared to the adult one. Finally, we analyzed the electrophoretic properties of collagens by SDS-PAGE method. For this, 5 μg of either adult or old rat type I collagen were analyzed on 5% polyacrylamide gels under reducing conditions. As can be seen in Figure 1E, both collagens exhibited the two characteristic chains α1 and α2 of native type I collagen. For old collagen, both chains migrated slower than in the case of adult collagen indicating a higher density of these chains in old collagen. The intensity of both chain bands was lower in old collagen than in the adult one. This could be due to an increased amount of higher molecular weight polymers in old collagen [18].

### Effect of aging on cell proliferation

**Figure 2:**
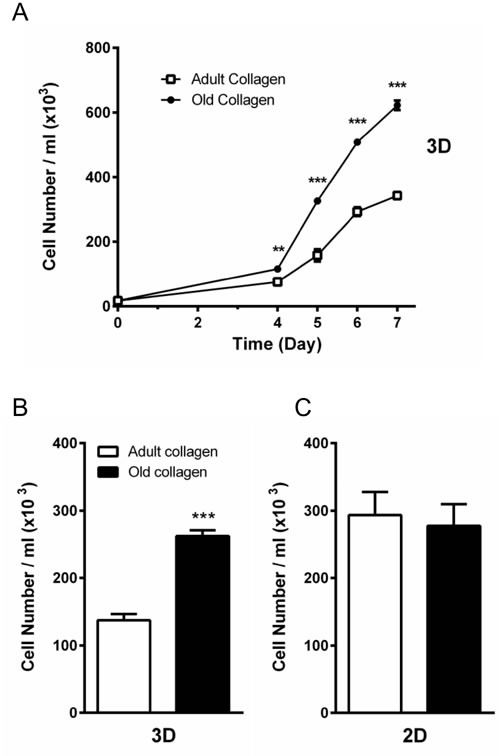
Effect of collagen aging on HT-1080 cell proliferation in 2D and 3D matrices **A.** HT-1080 cells were seeded in adult and old type I collagen 3D matrices at a density of 1.5 × 10^4^ cells/ml. After 4, 5, 6 and 7 days of culture, cell density was evaluated by phase contrast microscopy. **B.** HT-1080 cells were seeded in adult and old type I collagen 3D matrices at a density of 1.5 × 10^4^ cells/ml. After 5 days of culture, cell density was evaluated. **C.** HT-1080 cells were seeded on adult and old type I collagen 2D coating at a density of 1.5 × 10^4^ cells/ml. After 5 days of culture, cell density was evaluated by phase contrast microscopy. Values represent the mean ± S.E.M. of three independent experiments (**p < 0.01, ***p < 0.001).

We then examined whether contact with adult vs. old collagen gels differentially influenced the proliferative responses of the HT-1080 cells. For this, HT-1080 cells were seeded in adult and old collagen 3D matrices and cell growth was evaluated up to 7 days of culture. As shown in Figure 2A, HT-1080 cells in old collagen exhibited a significantly higher proliferation rate as early as day 4 of culture (*p* < 0.01). This difference in cell proliferation markedly increased up to day 7 (*p* < 0.001). We then compared the cell proliferation after 5 days of culture, in a 3D collagen matrix vs. 2D collagen coating. As shown in Figure 2B and 2C, the differential cell proliferation was only observed in 3D. In order to demonstrate the generality of this finding, we analyzed proliferation of A204 sarcoma cells in adult and old collagen 3D matrices. As shown in the supplementary data 1A, A204 cells exhibited also a significantly higher proliferation rate in old collagen when compared to the adult one. Taken together, these data indicate that collagen aging promotes HT-1080 cell proliferation, and that this process only occurs in a 3D environment.

### AGE receptor is not involved in the increased cell proliferation

**Figure 3:**
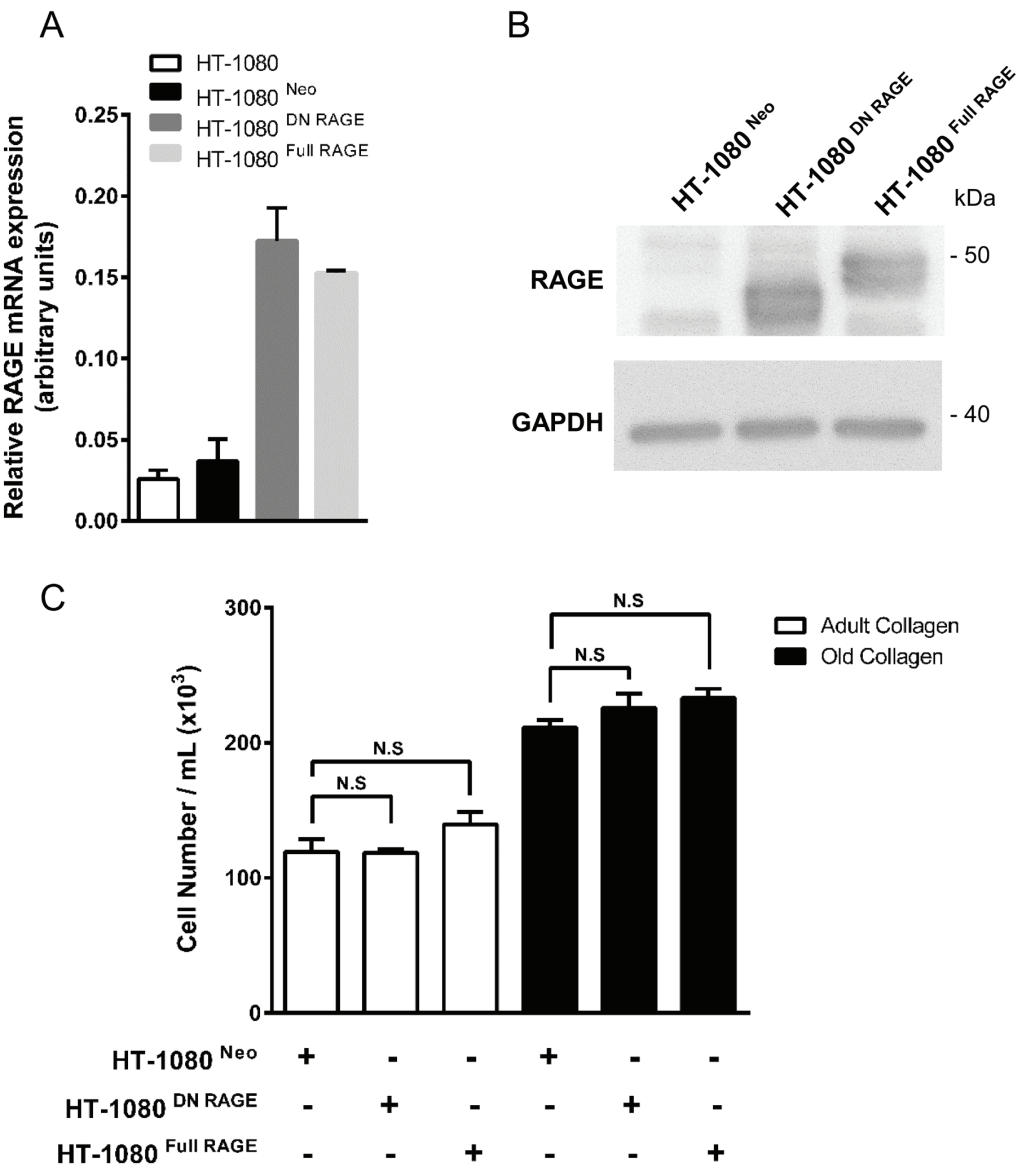
RAGE expression in parental and RAGE-transfected HT-1080 cells, and effect of collagen aging on cell proliferation Parental HT-1080 and HT-1080 cells stably transfected with empty vector (HT-1080 ^Neo^), human full length RAGE (HT-1080 ^Full RAGE^) and dominant negative RAGE (HT-1080 ^DN RAGE^) were cultured on plastic. **A.** RAGE transcripts contents in each cell lines were evaluated by real-time quantitative polymerase chain reaction. **B.** RAGE protein expression was analyzed by western blot. Glyceraldehyde 3-phosphate deshydrogenase (GAPDH) was used as a loading control. **C.** Parental and transfected HT-1080 cells were seeded in adult and old type I collagen 3D matrices at a density of 1.5 × 10^4^ cells/ml. After 5 days of culture, cell density was evaluated by phase contrast microscopy. Data shown are representative of three independent experiments (N.S. = not significant).

We studied the possible involvement of AGEs and their receptor RAGE in the regulation of cell proliferation. Indeed, AGE/RAGE axis has been shown to modulate tumor cell growth [19]. First we analyzed the expression of the RAGE mRNA using q-PCR. As shown in Figure 3A, RAGE was poorly expressed in HT-1080 cells. In order to exclude the role of RAGE in the increase of cell proliferation in old collagen, cell proliferation was analyzed in HT-1080 cells stably transfected with empty vector (HT-1080 ^Neo^), human full length RAGE (HT-1080 ^Full RAGE^) and dominant negative RAGE (HT-1080 ^DN RAGE^) [20]. As shown in Figure 3B and 3C, cell proliferation of HT-1080 ^Neo^ and HT-1080 ^DN RAGE^ cells increased in old collagen when compared to the adult one. This increase was similar to that observed in parental HT-1080 cells. Expression of full RAGE in HT-1080 cells (HT-1080 ^Full RAGE^) did not induce any significant increase in cell proliferation in old collagen when compared to the other cell lines. Altogether, these data indicate that RAGE is not involved in the increase of cell proliferation in old collagen.

### DDR2 but not β1 integrin is involved in the regulation of HT-1080 cell proliferation

**Figure 4:**
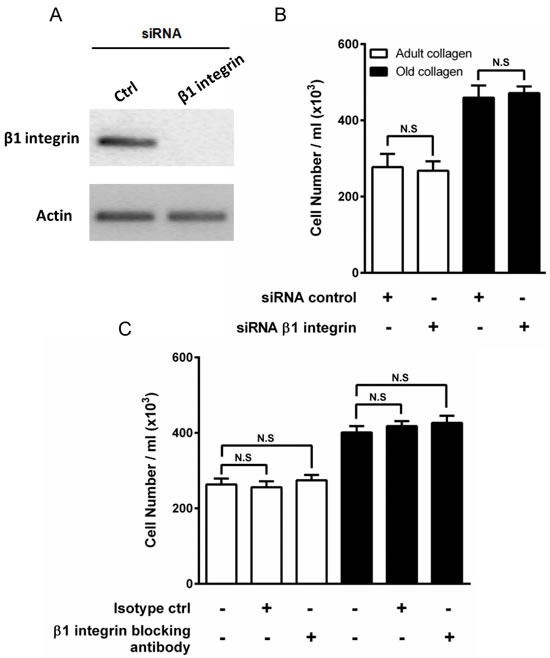
Effect of β1 integrin inhibition on HT-1080 cell proliferation **A.** HT-1080 cells were transfected with control siRNA (Ctrl) or β1 integrin siRNA. β1 integrin expression was analyzed by RT-PCR. Actin was used as a control. HT-1080 cells were seeded in adult and old type I collagen 3D matrices at a density of 5 × 10^4^ cells/ml, with or without **B.** siRNA directed against β1 integrin, or **C.** blocking antibody against β1 integrin (10 μg/ml). After 5 days of culture, cell density was evaluated by phase contrast microscopy. Values represent the mean ± S.E.M. of three independent experiments (N.S. = not significant).

A number of different transmembrane collagen receptors have been identified, with the integrin family being the best studied [21]. The primary collagen binding integrins are the α1β1 and α2β1 heterodimers [22]. Since we had previously demonstrated by flow cytometry that HT-1080 cells express β1 integrin [23], we investigated whether this integrin mediates the regulation of HT-1080 cell proliferation. siRNA was used to deplete β1 integrin in HT-1080 (Figure 4A), and cell proliferation was assessed in adult and old collagen. Figure 4B shows that β1 integrin depletion did not affect HT-1080 cell proliferation in both collagens, when compared to cells treated with control siRNA. Since cell proliferation was evaluated after 5 days culture, we analyzed β1 integrin mRNA expression using q-PCR after 2 and 5 days culture in 3D matrices. As shown in the supplementary data 2A, β1 integrin mRNA expression substantially decreased after 2 days culture. This expression was moderately decreased after 5 days culture. Preventing the involvement of integrin and collagen by using a blocking antibody against β1 integrin did not however modify cell proliferation in adult and old collagens (Figure 4C). Altogether, these data indicate that β1 integrin is not involved in the increase of HT-1080 cell proliferation in aged collagen.

**Figure 5:**
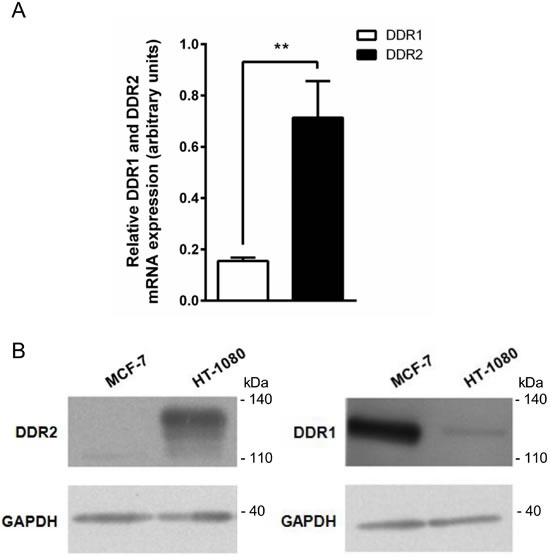
DDR1 and DDR2 expression in HT-1080 and MCF-7 cells HT-1080 and MCF-7 cells were cultured on plastic. **A.** DDR2 and DDR1 transcripts contents in HT-1080 cells were evaluated by real-time quantitative polymerase chain reaction. **B.** DDR2 (left panel) and DDR1 (right panel) protein expression was analyzed by western blot. Glyceraldehyde 3-phosphate dehydrogenase (GAPDH) was used as a loading control. Data shown are representative of three independent experiments (**p < 0.01, ***p < 0.001).

DDR1 and DDR2 are collagen-binding receptors belonging to the receptor tyrosine kinase (RTK) family. To determine whether these receptors play a role in the regulation of HT-1080 cell proliferation, we first evaluated DDR1 and DDR2 expression in these cells. Quantitative PCR analysis shows in Figure 5A that DDR2 mRNA was preferentially expressed in HT-1080 cells. These data were confirmed by Western blot, showing that DDR2 was significantly more expressed than DDR1 in HT-1080 cells (Figure 5B). MCF-7 cells were used here as a negative control for DDR2 [24] and a positive control for DDR1 [25].

**Figure 6:**
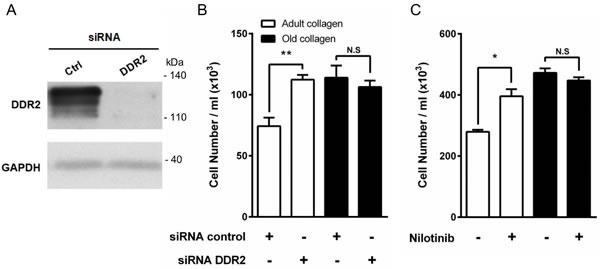
Effect of DDR2 inhibition on HT-1080 cell proliferation **A.** HT-1080 cells were transfected with control siRNA (Ctrl) or DDR2 siRNA. DDR2 expression was analyzed by immunoblotting. GAPDH was used as a loading control. **B.** HT-1080 cells were seeded in adult and old type I collagen 3D matrices at a density of 1.5 × 10^4^ cells/ml, with or without siRNA directed against DDR2. After 5 days of culture, cell density was evaluated by phase contrast microscopy. **C.** HT-1080 cells were seeded in adult and old type I collagen 3D matrices at a density of 5 × 10^4^ cells/ml, with or without DDR2 inhibitor nilotinib at 100 nM. After 5 days of culture, cell density was evaluated by phase contrast microscopy. Values represent the mean ± S.E.M. of three independent experiments (*p < 0.05, **p < 0.01, N.S. = not significant).

The involvement of DDR2 in HT-1080 cell proliferation was then investigated. To this end, DDR2 siRNA was used to knockdown DDR2 (Figure 6A). Figure 6B shows that DDR2 depletion promotes cell proliferation in adult collagen but not in the old one. As previously performed for β1 integrin siRNA (supplementary data 2A), we analyzed also DDR2 mRNA expression using q-PCR after 2 and 5 days culture in 3D matrices. As shown in the supplementary data 2B, DDR2 mRNA expression highly decreased after 2 days culture. However, this expression increased to the same level observed in control cells after 5 days culture. These data were confirmed by using nilotinib, a well-known inhibitor of DDR2 kinase activity [26]. Nilotinib at non-toxic concentration (100 nM) was able to restore cell proliferation in adult collagen to a level similar to that observed in the old one (Figure 6C). As nilotinib may inhibit other kinases in addition to DDR2, we investigated whether nilotinib was able to restore cell proliferation in HT-1080 cells expressing the gatekeeper DDR2 form (T654I), which is not sensitive to nilotinib [27, 28]. As shown in the supplementary data 3A, HT-1080 cells expressed the same level of DDR2 protein when transfected with wild-type and gatekeeper form of DDR2. In the presence of nilotinib, the proliferation did not increase in cells expressing gatekeeper DDR2 form when compared to those expressing the wild-type form of the receptor (Supplementary Data 3A). These data suggest that DDR2 regulates differentially HT-1080 cell proliferation in adult and old 3D collagen matrices.

### DDR2 is highly activated in adult collagen

**Figure 7:**
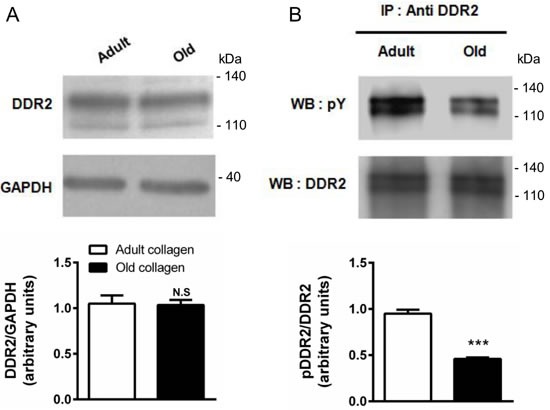
Effect of collagen aging on DDR2 expression and activation HT-1080 cells were serum starved during 12 hours, then cultured 6 hours in adult and old type I collagen 3D matrices. **A.** Western blot analysis was performed using anti-DDR2 specific antibody. The histogram shows the ratio of DDR2 expression relative to the loading control GAPDH. **B.** Immunoprecipitation was performed using anti-DDR2 specific antibody, and DDR2 activation was measured by western blot using anti-phosphotyrosine specific antibody. The histogram shows the ratio of pDDR2 expression relative to DDR2. Values represent the mean ± S.E.M. of three independent experiments (***p < 0.001, N.S. = not significant).

Several reports suggest that DDR2 is a tyrosine kinase receptor with the unique ability to be activated by fibrillar collagen [11]. Contrary to other classical growth factor-activated RTKs which display rapid and transient activation, DDR2 exhibit delayed and sustained phosphorylation upon binding to collagen. Its expression in HT-1080 cells in adult and old collagen was first analyzed. Figure 7A shows that DDR2 expression levels were similar in both collagens. In order to investigate whether DDR2 was differentially activated in adult and old collagen, HT-1080 cells were seeded in collagen 3D matrices. DDR2 was immunoprecipitated from cell lysates and its phosphorylated form was detected by immunoblotting. As shown in Figure 7B, DDR2 phosphorylation was 2-fold higher in adult collagen when compared to the old one (*p* < 0.001). As for cell proliferation, we then analyzed the activation of DDR2 in A204 sarcoma cells in collagen 3D matrices. As shown in the supplementary data 1B, DDR2 phosphorylation was also higher in adult collagen when compared to the old one. Since the cell proliferation rate was similar on adult and old 2D collagen coating, we investigated whether DDR2 activation was similar or not in these conditions. As shown in the supplementary data 4, DDR2 phosphorylation in the case of adult 2D collagen coating was similar to that observed in the case of the older one. This suggests that the 3D matrix model is crucial for the differential DDR2 activation with type I collagen aging.

### The primary DDR2 downstream target SHP-2 is highly activated in adult collagen

**Figure 8:**
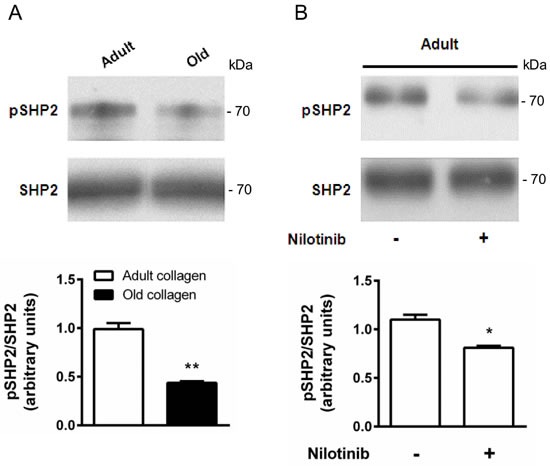
Effect of collagen aging on SHP-2 activation **A.** HT-1080 cells were serum starved during 12 hours, then cultured 6 hours in adult and old type I collagen 3D matrices. Western blot analysis was performed using anti-pSHP-2 and SHP-2 specific antibodies. The histogram shows the ratio of pSHP-2 expression relative to SHP-2. **B.** HT-1080 cells were serum starved during 12 hours, then cultured 6 hours in adult type I collagen 3D matrices, with or without 100 nM of nilotinib. Western blot analysis was performed using anti-pSHP-2 and SHP-2 specific antibodies. The histogram shows the ratio of pSHP-2 expression relative to total SHP-2. Values represent the mean ± S.E.M. of three independent experiments (*p < 0.05, **p < 0.01).

DDR2 has been shown to phosphorylate the tyrosine phosphatase SHP-2 when activated with fibrillar type I collagen [29]. To elucidate whether SHP-2 is also involved as a downstream effector of DDR2 in our model, HT-1080 cells were seeded in adult and old collagen matrices for 6 hours. Then, Western blotting experiments were performed in order to analyze SHP-2 expression and Tyr-542 phosphorylation. Figure 8A shows that SHP-2 expression was similar for both collagens, whereas SHP-2 phosphorylation was increased 2-fold in adult collagen compared to the old one. As shown in the supplementary data 1C, SHP-2 phosphorylation also increased in A204 cells in adult collagen. When DDR2 expression was suppressed in HT-1080 cells using siRNA, SHP-2 phosphorylation decreased in adult collagen (supplementary data 5A). In adult collagen, SHP-2 phosphorylation was inhibited by approximatively 30% when HT-1080 cells were treated with 100nM nilotinib (Figure 8B). In order to show that nilotinib induced this effect by inhibiting specifically DDR2, we investigated whether SHP-2 phosphorylation was inhibited by nilotinib in gatekeeper DDR2 expressing cells. As shown in the supplementary data 3B, SHP-2 phosphorylation level was similar when cells were treated or not with nilotinib. Taking into account that nilotinib was able to increase cell proliferation in adult collagen (Figure 6), these data suggest that DDR2 downregulates cell proliferation in adult collagen by SHP-2 downstream activation.

### JAK2 phosphorylation is downregulated in adult collagen

**Figure 9:**
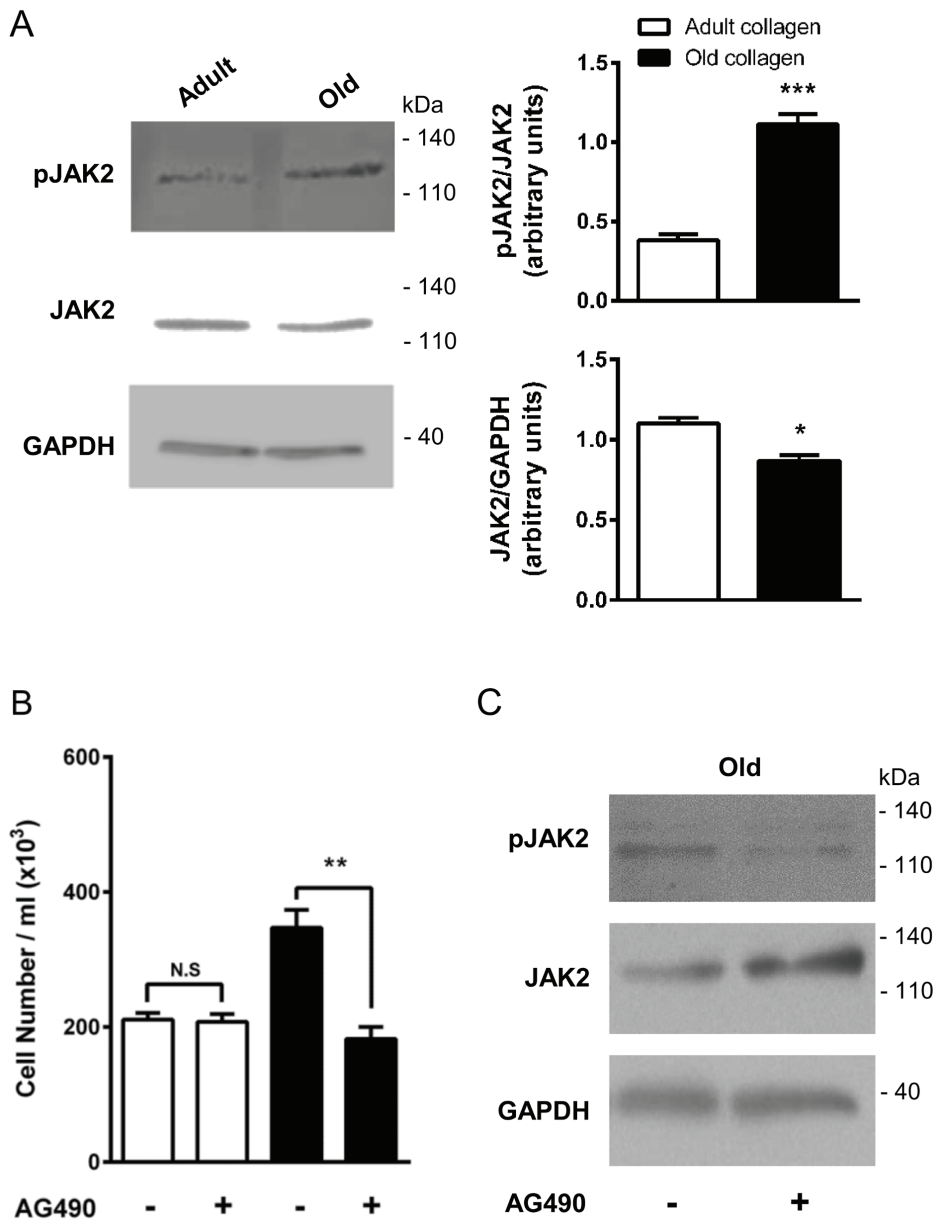
Effect of collagen aging on JAK2 activation and HT-1080 cell proliferation HT-1080 cells were cultured 5 days in adult and old type I collagen 3D matrices. **A.** Western blot analysis was performed using pJAK2 and JAK2 specific antibodies. The histogram shows the ratio of pJAK2 and JAK2 expression relative to JAK2 and GAPDH respectively. **B.** HT-1080 cells were seeded in adult and old type I collagen 3D matrices at a concentration of 1.5 × 10^4^ cells/ml, with or without 10 μM of the JAK2 inhibitor AG490. After 5 days of culture, cell density was evaluated by phase contrast microscopy and **C.** western blot analysis was performed using pJAK2 and JAK2 specific antibodies. Values represent the mean ± S.E.M. of three independent experiments (*p < 0.05, **p < 0.01, ***p < 0.001).

The Tyr-1007 site of JAK2 has been reported to be dephosphorylated by tyrosine phosphatase SHP-2 [30]. In order to determine whether SHP-2 activation in the presence of adult collagen is able to downregulate JAK2 phosphorylation, JAK2 expression and phosphorylation were analyzed by Western blott. Figure 9A shows that JAK2 phosphorylation level was 3-fold lower in adult collagen than in the old one. As shown in supplementary data 1C, JAK2 phosphorylation was also decreased in A204 cells in adult collagen. It is important to note that in both cell lines, JAK2 expression was significantly decreased in the presence of old collagen. In order to investigate whether kinase function of DDR2 was involved in the activation of JAK2, we analyzed JAK2 phosphorylation in wild-type and gatekeeper DDR2 expressing cells in the presence or not of 100 nM nilotinib. As shown in supplementary data 3B, nilotinib was able to increase JAK2 phosphorylation in wild-type DDR2 expressing cells but not in gatekeeper ones. When SHP-2 expression was suppressed in HT-1080 cells using siRNA, JAK2 phosphorylation increased in adult collagen (supplementary data 6). To establish whether JAK2 phosphorylation was involved in the increase of cell proliferation in old collagen, we analyzed cell proliferation in the presence of the p-JAK2 inhibitor AG490 in both collagens. At 10 μM concentration, AG490 was able to decrease HT-1080 cell proliferation in old collagen but not in the adult one (Figure 9B). To demonstrate that the inhibition of cell proliferation in the presence of AG490 was due to a decrease in JAK2 activation, JAK2 phosphorylation was analyzed after AG490 treatment. As shown in Figure 9C, AG490 induced a decrease in JAK2 phosphorylation.

### ERK1/2 phosphorylation is downregulated by adult collagen

**Figure 10:**
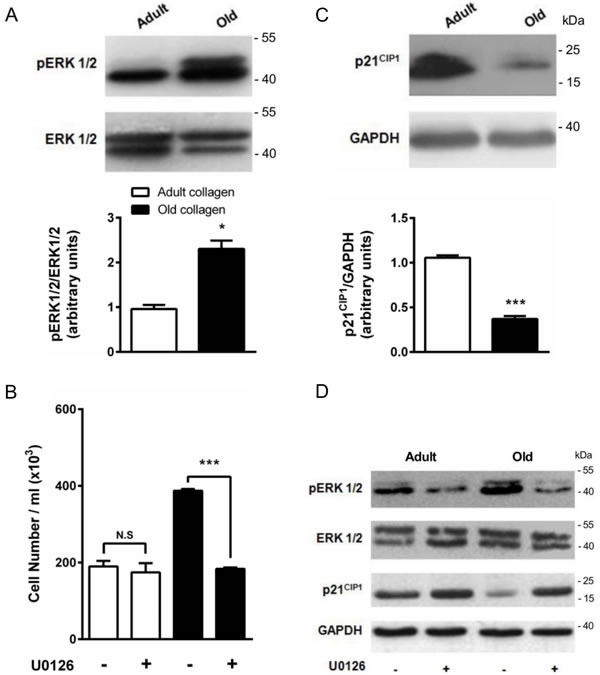
Effect of collagen aging on ERK1/2 activation and p21^CIP1^ expression **A.** ERK1/2 western blot analysis of HT-1080 cells after 5 days culture in adult and old type I collagen 3D matrices. The histograms show the ratio of pERK1/2 expression relative to total ERK1/2. **B.** Effect of ERK1/2 inhibitor U0126 on cell proliferation. HT-1080 cells were seeded in adult and old type I collagen 3D matrices at a density of 1.5 × 10^4^ cells/ml, with or without 5 μM U0126. After 5 days of culture, cell density was evaluated by phase contrast microscopy. **C.** p21^CIP1^ western blot analysis of HT-1080 cells after 5 days culture in adult and old type I collagen 3D matrices. The histograms show the ratio of p21^CIP1^ expression relative to GAPDH. **D.** Effect of U0126 on pERK1/2 and p21^CIP1^ expression. HT-1080 cells were seeded in adult and old type I collagen 3D matrices at a density of 1.5 × 10^4^ cells/ml, with or without 5 μM U0126. After 5 days of culture, western blot analysis was performed using pERK1/2 and p21^CIP1^ specific antibodies. Values represent the mean ± S.E.M. of three independent experiments (*p < 0.05, ***p < 0.001).

**Figure 11:**
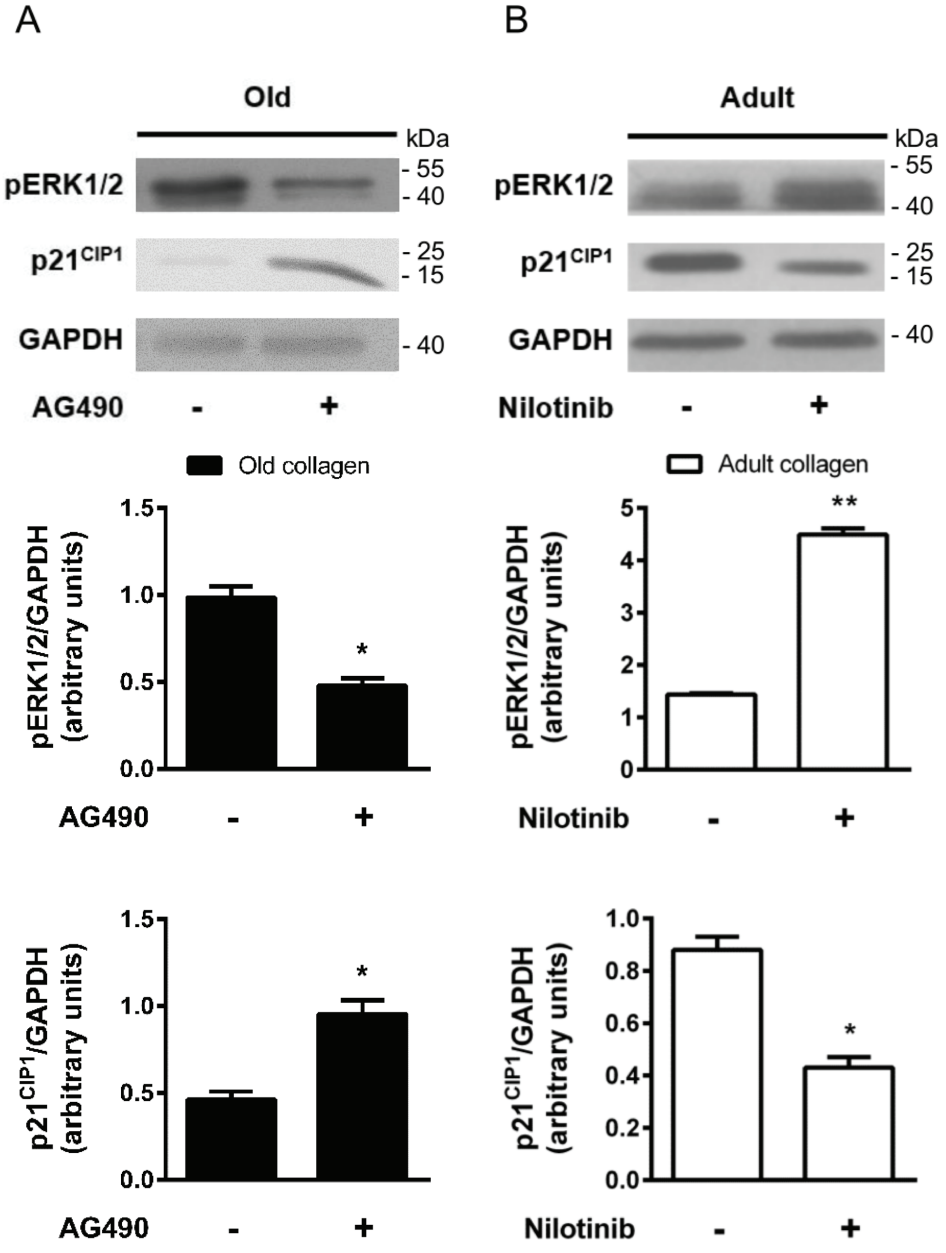
Effect of DDR2 and JAK2 inhibition on ERK1/2 activation and p21^CIP1^ expression **A.** HT-1080 cells were cultured 5 days in old type I collagen 3D matrices, with or without 10 μM of AG490. Western blot analysis was performed using pERK1/2 and p21^CIP1^ specific antibodies. The histograms show the ratio of pERK1/2 and p21^CIP1^ expression relative to GAPDH. **B.** HT-1080 cells were cultured 5 days in adult type I collagen 3D matrices, with or without 100 nM of nilotinib. Western blot analysis was performed using pERK1/2 and p21^CIP1^ specific antibodies. The histograms show the ratio of pERK1/2 and p21^CIP1^ expression relative to GAPDH. Values represent the mean ± S.E.M. of three independent experiments (*p < 0.05, **p < 0.01).

ERK1/2 pathway has been shown to be phosphorylated by p-JAK2 [31] and is known to promote cell proliferation in various cell models [32]. To determine whether ERK1/2 was differentially activated in adult and old collagen, ERK1/2 phosphorylation was analyzed by Western blot. Figure 10A indicates that ERK1/2 phosphorylation was 2-fold lower in adult collagen when compared to the old one (*p* < 0.05). As shown in the supplementary data 1D, ERK1/2 phosphorylation was also decreased in A204 cells in adult collagen. In order to verify whether ERK1/2 function upregulates cell proliferation in old collagen, cells were treated with the ERK1/2 phosphorylation inhibitor U0126. Figure 10B shows that U0126 was able to decrease cell proliferation in old collagen to a level close to that of the adult one. In order to verify if there is a relationship between JAK2 activity and ERK1/2 phosphorylation in old collagen, cells were treated with the JAK2 inhibitor AG490 before analysis of ERK1/2 phosphorylation. As shown in Figure 11A, ERK1/2 phosphorylation was 2-fold lower when cells were treated with AG490 in old collagen. To establish a link between DDR2 activation and ERK1/2 pathway inhibition, DDR2 expression was suppressed in HT-1080 cells using siRNA and ERK1/2 pathway was analyzed in adult collagen. ERK1/2 phosphorylation was increased (supplementary data 5B). ERK1/2 activation was also analyzed after nilotinib treatment in adult collagen. As shown in Figure 11B, ERK1/2 phosphorylation was increased 3-fold in the presence of nilotinib compared to control cells. In order to show that nilotinib induced this effect by inhibiting specifically DDR2, we investigated whether ERK1/2 phosphorylation was increased by nilotinib in gatekeeper DDR2 expressing cells. As shown in the supplementary data 3C, ERK1/2 phosphorylation level was similar when cells were treated or not with nilotinib.

### p21^CIP1^ expression is upregulated in adult collagen

The cyclin inhibitor p21^CIP1^ is well known to downregulate cell cycle progression and to inhibit cell proliferation. As shown in Figure 10C, p21^CIP1^ expression was 3-fold higher in adult collagen relative to the old one. As shown in the supplementary data 1D, p21^CIP^ expression also increased in A204 cells in adult collagen. In order to determine whether this negative regulatory control of p21^CIP1^ expression involved ERK1/2 phosphorylation [33], cells were treated with the ERK1/2 phosphorylation inhibitor U0126. Figure 10D shows that U0126 was able to increase p21^CIP1^ expression in old collagen. In order to verify if there is a relationship between JAK2 activity and p21^CIP1^ expression in old collagen, cells were treated with the JAK2 inhibitor AG490 before analysis of p21^CIP1^ expression. As shown in Figure 11A, p21^CIP1^ expression was 2-fold higher when cells were treated with AG490 in old collagen. Finally, to establish a link between DDR2 activation and p21^CIP1^ expression, DDR2 expression was suppressed in HT-1080 cells using siRNA and p21^CIP1^ expression was analyzed in adult collagen. As shown in the supplementary data 5B, p21^CIP1^ expression was decreased. p21^CIP1^ expression was also analyzed after nilotinib treatment in adult collagen. As shown in Figure 11B, nilotinib induced a 2-fold decrease in p21^CIP1^ expression. As for ERK1/2, and in order to show that nilotinib induced this effect by inhibiting specifically DDR2, we investigated whether p21^CIP1^ expression was decreased by nilotinib in gatekeeper DDR2 expressing cells. As shown in the supplementary data 3C, p21^CIP1^ expression level was similar when cells were treated or not with nilotinib.

## DISCUSSION

The ECM, which was previously considered as a physical scaffold playing a role in tissue organization, is currently recognized as a key regulator of signaling pathways involved in cell and tissue functions. Several works have been reported on the dynamic interplay between type I collagen, the major component of ECM in interstitial tissue, and tumor [34]. To understand this cross-talk, previous published works have already shown that type I collagen is able to restrict proliferation of cancer cells [35-37]. Proliferative responses seemed to be related to 3D culture conditions [38, 39]. Thus, such 3D *in vitro* models provide a bridge between 2D cell culture and *in vivo* models. Several groups using 3D models demonstrated that tumor cell proliferation was restricted in the presence of type I collagen [7, 37, 40].

In this work, we aimed at analyzing the effect of collagen aging on HT-1080 tumor cell proliferation. We report evidence that proliferation of HT-1080 cells is higher in old collagen when compared to the adult one. This is in agreement with previously reported data on lung [5], and prostate [41] carcinomas. The culture model was crucial since the observed difference in cell proliferation occurred in 3D but not in 2D cell culture systems. Using siRNA strategy and blocking antibodies, we show that this regulation does not involve β1 integrin in 3D conditions. In addition, in our recent findings collagen 3D conditions induce a rapid and marked decrease in β1 integrin expression in HT-1080 cells [23]. This support the fact that β1 integrin is not involved in the differential regulation of cell proliferation. Using blocking antibodies and antagonists against RAGE, we also demonstrate that the AGE/RAGE axis is not involved. Consistently, HT-1080 cell treatment with glycated bovine serum albumin did not increase significantly cell proliferation (data not shown).

In the presence of adult collagen, DDR2 phosphorylation was significantly higher in adult collagen when compared to the old one. We further show that downregulation of DDR2 expression and inhibition of its kinase function by nilotinib induced an increase in HT-1080 cell proliferation to a level similar to that of the old one. To our knowledge, we show for the first time a differential DDR2 activation in adult and old collagen suggesting the involvement of DDR2 in the regulation of cell proliferation by collagen aging. DDR2 is well known to be activated by fibrillar type I collagen [15] and this property is crucial in the down-regulation of tumor cell proliferation by this receptor [22]. Interestingly, our group has recently observed detrimental changes in fibrillar type I collagen properties with aging [6, 17] that could explain the loss of DDR2 activation by old collagen resulting from impaired DDR2/collagen interactions.

The tyrosine phosphatase SHP-2, a specific downstream effector of DDR2 [29], is more phosphorylated in the presence of adult collagen when compared with the old one. This highly correlates with the differential activation of DDR2 observed between the two collagens. In addition, using nilotinib, we confirm that DDR2 kinase activity is required for SHP-2 phosphorylation (Figure 8), as described in lung cancer expressing DDR2 with mutations in the kinase domain [29]. The phosphorylated Tyr-1007 of JAK2, which is necessary for its kinase activity [42] has been proved to be a target for SHP-2 [30]. Here, we show that the phosphorylation level of JAK2 Tyr-1007 was lower in adult collagen when compared to the old one. This suggests that DDR2-mediated SHP-2 activation is a critical step in the down-regulation of JAK2 activity and consequently in cell growth suppression.

We demonstrate that ERK1/2 phosphorylation, known to promote cell proliferation [32], is lower in the presence of adult collagen. ERK1/2 has been shown to phosphorylate p21^CIP1^ which is subsequently exported from the nucleus and ubiquitinated before degradation in the proteasome [43]. Consistently, we find an overexpression of p21^CIP1^ in the presence of adult collagen associated with a decrease in ERK1/2 phosphorylation. We also show that DDR2 inhibition in adult collagen induces an increase in ERK1/2 phosphorylation and downregulation of p21^CIP1^. In addition, JAK2 inhibition in old collagen induces a decrease in ERK1/2 phosphorylation and an increase in p21^CIP1^ expression. This establishes a link between the ERK1/2-p21^CIP1^ and DDR2-JAK2 axes in the regulation of HT-1080 tumor cell growth.

**Figure 12:**
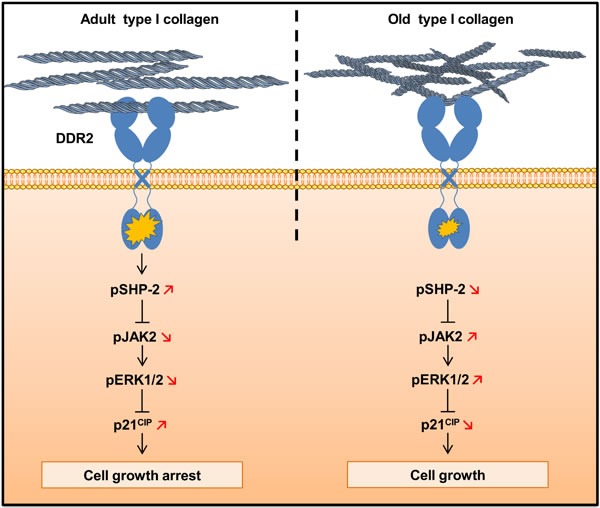
Schematic drawing of DDR2-induced cell growth regulation by type I collagen aging

In conclusion, we show in agreement with previously reported data [5, 41] that biologically aged type I collagen has the capacity to increase the proliferative behavior of human tumor cells in a 3D context. We identify for the first time DDR2 as a key matrix aging sensor responsible for the regulation of this process. As summarized in Figure 12, the regulation of cell proliferation by the collagen/DDR2 axis involves a differential activation of SHP-2, JAK2/ERK1/2 signaling pathways, and p21^CIP1^ expression. In addition, our study suggests that the impact of matrix aging has to be taken into account when designing *in-vitro* experiments to understand cell/ECM interactions, and to evaluate their consequences on tumor cell responses. Such age-dependent processes may also contribute to better address the higher proliferative tumors behavior observed for elderly patients [44, 45].

## MATERIALS AND METHODS

### Cell lines

The human fibrosarcoma cell line HT-1080 (CCL-121) and the human breast adenocarcinoma cell line MCF-7 (HTB-22) were purchased from the American Type Culture Collection (ATCC, Rockville, MD, USA). HT-1080 cells stably transfected with empty vector (HT-1080 ^Neo^), human full length RAGE (HT-1080 ^Full RAGE^) and dominant negative RAGE (HT-1080 ^DN RAGE^) were obtained from Dr. Takeuchi A. (Kanazawa Medical University, Uchinada, Japan) [20]. All these cell lines were cultured in MEM with Earle salts and Glutamax I (Invitrogen, Cergy-pontoise, France) supplemented with 10% fetal bovine serum, (Invitrogen) and 1% penicillin-streptomycin (Invitrogen). RAGE transfected HT-1080 cells were cultured in the presence of 750 μg/mL G418 (Roche Applied Science, Mannheim, Germany). Cultures were maintained at 37°C in a humidified atmosphere containing 5% CO_2_ (v/v). Cells were routinely passaged at preconfluency using 0.05% trypsin, 0.53 mM EDTA (Invitrogen) and screened for the absence of mycoplasma using PCR methods.

### Preparation and characterization of type I collagen

Fibrillar and non-pepsinized collagen type I was extracted from tail tendons of 2 months (adult) and 2 years old (old) rats, and prepared as already described [46]. Spectrofluorimetric analysis was performed on adult and old collagen solubilized at 2 mg/ml in 18 mM acetic acid (v/v) to detect AGEs-specific fluorescence using a spectrofluorimeter (Shimadzu model RF-500) at λ_ex_ = 380 nm and λ_em_ = 440 nm. AGE-modified BSA was used as a standard. Pentosidine and CML concentrations were measured using LC/MS/MS. One mg of collagen was hydrolyzed at 110°C during 18 hours in the 6 M HCl, and hydrolysates were evaporated to dryness under nitrogen stream. Dried hydrolyzates were then resuspended in 100 μl of 125 mM ammonium formate, prior to LC-MS/MS analysis with a gradient composed of 5 mM ammonium formate (pH 2.9) as mobile phase A and 100% acetonitrile as mobile phase B. Detection was performed using an API4000 system (ABSciex, France) in positive-ion mode with an elestrospray ionisation (ESI) source. Cross-links quantification was performed by ion exchange chromatography (Hitachi L-8800). Collagens hydrolysates were prepared as described above. Dried hydrolyzates were then resuspended in 100 μl of a buffer composed of (lithium 13.86 mM, lithium citrate 55 mM, citric acid 207 mM, ethanol 6% (v/v), thiodiglycol 1% (v/v)) pH 2.8. The chromatography was performed according to the manufacturer's instructions. The two enzymatic cross-links hydroxylysylpyridinoline and lysylpyrodinoline were quantified and expressed as μmol/mol collagen. Electrophoretic properties of aged collagen were estimated by 5% sodium dodecyl sulfate-polyacrylamide gel electrophoresis (SDS-PAGE), under denaturing conditions (4 min at 90°C). Gels were stained with Coomassie Brillant Blue R250.

### 2D and 3D cell culture

The effects of the aged type I collagen on HT-1080 cell proliferation were studied using 24-well plates. For 2D cell culture, each well was coated by adding 250 μl of native or modified collagens solubilized in 0.018 M acetic acid at a concentration of 38 μg/ml. Then, coated substrates were dried overnight at room temperature under sterile conditions and rinsed once in PBS (Invitrogen) before cell plating. HT-1080 cells were seeded on the coated surfaces at a concentration of 1.5 × 10^4^ cells/well (1 ml/well). For 3D cell culture, 1.5 × 10^4^ HT-1080 cells were resuspended in 100 μl fetal bovine serum and mixed with a solution containing 100 μl MEM 10X (Sigma-Aldrich, St. Quentin-Fallavier, France), 100 μl NaHCO_3_ 0.26 M, 100 μl H_2_O, 90 μl NaOH 0.1 M, 10 μl glutamine 200 mM and 500 μl collagen 3 mg/ml. This solution was deposited in 24-well plates (1 ml/well). After polymerization at 37°C during 10 min, gels were recovered by 1 ml MEM 10% foetal bovine serum and 3D cultures were incubated during 4 to 7 days. Then, medium recovering 3D cultures was eliminated, and gels were digested by collagenase P at 3 mg/ml (Roche, Meylan, France), cell viability and density were determined by phase contrast microscopy. During these experiments, the covering culture medium was not renewed.

### Blocking antibody and pharmalogical inhibitors

In some experiments, cells were treated with blocking antibodies or different pharmacological inhibitors. Blocking antibodies were preincubated with the cells during 30 min at room temperature, at the following concentration: β1 integrin antibody (10 μg/ml, MAB2253Z, Millipore, St. Quentin en Yvelines, France). Pharmacological inhibitors were added in the gels at the indicated concentrations: U0126 (5 μM, Promega, Charbonnière-les-bains, France), AG490 (10 μM, Santa Cruz Biotechnology) and nilotinib (100 nM, Selleckchem, Souffelweyersheim, France). Cells were then embedded in collagen 3D matrices, as described above.

### siRNA transfections

siRNA oligonucleotides were transfected in HT-1080 cells with Lipofectamine RNAiMax (Invitrogen) according to the manufacturer's instructions. Pools of 3 target-specific 19-25 nucleotides DDR2 siRNA (sc-39922) and β1 integrin siRNA (sc-35674) were purchased from Santa Cruz Biotechnology, and were used at 50 nM. Negative control siRNA (1027310) was purchased from Qiagen (Courtaboeuf, France). Cells were allowed to grow 24 hours after transfection before use. DDR2 and β1 integrin extinction were controlled by western blotting and RT-PCR respectively, then cells were seeded in type I collagen 3D matrices as described above.

### Western blotting

Cells were lyzed with RadioImmunoPrecipitation Assay (RIPA) buffer (Thermo Fisher Scientific, Villebon sur Yvette, France). Cell lysates were clarified by centrifugation at 14000 × g at 4°C for 15 min. For western blotting, proteins were separated by SDS-PAGE gels and transferred to a nitrocellulose membrane. Then, membranes were blocked with Tris buffered saline (TBS) (0.02 M Tris-HCl, 0.137 M NaCl, pH 7.4) containing 0.1% Tween (TBS-T) and 5% non-fat dry milk at room temperature during 1 hour and incubated overnight at 4°C with the following primary antibodies: anti-phospho-JAK2 (Santa Cruz Biotechnology), anti-GAPDH, anti-DDR1, anti-phospho-SHP2, anti-SHP2, anti-JAK2, anti-phospho-ERK1/2, anti-ERK1/2, anti-p21^CIP1^ (Cell signaling Technology, Saint Quentin Yvelines, France), anti-DDR2 (R&D systems, Lille, France), anti-RAGE (GeneTex, Irvine, CA). Membranes were washed with TBS-T and incubated with the corresponding peroxidase conjugated secondary antibody at room temperature for 1 hour. Chemiluminescent detection was realized by using an ECL Prime Kit (GE Healthcare, Orsay, France).

### Immunoprecipitation

HT-1080 cells were washed with DPBS and incubated overnight in serum-free media prior 6 hours of stimulation in adult and old type I collagen 3D matrices. Cells were then lysed with RIPA buffer, and cell lysates were clarified by centrifugation at 14000 × g at 4°C for 15 min. Then 300 μg of whole-cell extracts was immunoprecipitated with anti-DDR2 (1:100, Santa Cruz Biotechnology) at 4°C for 12 hours and then bound to protein G agarose beads (GE Healthcare) and finally washed three times with TBS. The proteins were separated by SDS-PAGE, and the immunoprecipitates were blotted with anti-phosphotyrosine 4G10 antibodies (Millipore). The blots were then stripped using a stripping buffer (100 mM 2-mercaptoethanol, 2% SDS, 63 mM Tris-HCl pH 6.8) and re-probed with anti-DDR2 (R&D systems) antibody.

### Quantitative RT-PCR

Cells were seeded in 6-well plates at a density of 1.5 × 10^4^ cells/well. After 3 days, culture media were removed and the cells were washed two times with cold DPBS. Total RNA purification was performed with the RNeasy Mini Kit (Qiagen) and 1 μg of RNA was converted to cDNA by reverse transcription using the Maxima First Strand cDNA Synthesis kit (Thermo Fisher Scientific), and a PCR MasterCycler^®^ (Eppendorf, Montesson, France). Then real-time PCR was performed using a Maxima SYBR GREEN/ROX qPCR Master Mix (Thermo Fisher Scientific) on the Stratagene Mx3005P qPCR detection system (Agilent Technologies, Les Ulis, France). Polymerase chain reaction conditions were 15 min at +95°C, followed by 35 cycles each consisting of 15 s at +95°C (denaturation) and 30 s at +60°C (annealing/extension). Results were standardized to the eEF1a1 gene expression by calculating ΔCt using the formula ΔCt = (Ct_gene of interest_ - Ct_eEF1A_). Gene expression was represented as 2^−ΔCt^. The forward primer for DDR1 transcript was 5′-TGCTCTCCAATCCAGCCTAC-3′ and the reverse primer was 5′-ATTATGCCGAGGCTGACATT-3′ with a 203 bp product. The forward primer for DDR2 transcript was 5′-GCGCCATGCAGGAGGTCTAG -3′ and the reverse primer was 5′-CCACTCTCATACACACATTCA-3′ with a 230 bp product. The forward primer for eEF1A transcript was 5′-CTGGAGCCAAGTGCTAACATGCC-3′ and the reverse primer was 5′-CCGGGTTTGAGAACACCAGTC-3′ with a 221 bp product. The forward primer for RAGE transcript was 5′-AAACATCACAGCCCGGATTG-3′ and the reverse primer was 5′- TCCGGCCTGTGTTCAGTTTCC-3′ with a 101 bp product. To verify β1 integrin silencing, a classical PCR amplification was carried out. The forward primer for β1 integrin transcript was 5′-TGCGAGTGTGGTGTCTGTAA-3′ and the reverse primer was 5′-AGGCTCTGCACTGAACACAT-3′ with a 118 bp product. The forward primer for actin transcript was 5′-GTGTGACGTGGACATCCGC-3′ and the reverse primer was 5′-CTGCATCCTGTCGGCAATG-3′ with a 91 bp product. The resulting PCR products were visualized by ultraviolet after staining with ethidium bromide.

### Statistical analysis

Data are presented as mean ± standard error of the mean (SEM). The values were analyzed with Student's t-test. p values lower than 0.05 were considered as significant. *p < 0.05; **p < 0.01; ***p < 0.001. Electrophoretic images were analyzed with ImageJ software.

## SUPPLEMENTARY MATERIALS FIGURES


