# Supplementary material for: Type I collagen aging impairs discoidin domain receptor 2-mediated tumor cell growth suppression
Source: Oncotarget. 2016 Apr 18;7(18):24908–27. doi: 10.18632/oncotarget.8795 (PMC5041879; doi:10.18632/oncotarget.8795)
Supplement: Supplementary file 1 [file oncotarget-07-24908-s001.pdf]

Type I collagen aging impairs discoidin domain receptor 2-mediated tumor cell growth suppression

Supplementary Material

Cell lines: The human sarcoma cell line A204 (HTB-82) were obtained from Dr. Trent J.C. from the University of Miami, FL, and cultured in MEM with Earle salts and Glutamax I (Invitrogen, Cergy-pontoise, France) supplemented with 10% fetal bovine serum, (Invitrogen) and 1% penicillin-streptomycin (Invitrogen). Cultures were maintained at 37˚C in a humidified atmosphere containing 5% CO_2 (v/v). Cells were routinely passaged at preconfluency using 0.05% trypsin, 0.53 mM EDTA (Invitrogen) and screened for the absence of mycoplasma using PCR methods.

DDR2 vectors and viral infections: Retroviruses were generated by co-transfection of p-Wzl-Blast vector with pCL-Ampho vector (Bio-Techne, Minneapolis, MN) [1, 2] into 293T retroviral packaging cell line from Dr. Rio M.C. (IGBMC, Illkirch, France) with Fugene 6 reagent (Roche, Mannheim, Germany). Viral particles were collected 48h after transfection. Viral particles supplemented with 10 µg/ml polybrene (Santa Cruz) and 20 mM Hepes were then incubated with HT-1080 cells. Selection was performed using 10 µg/ml blasticidine (Fisher Scientific, Illkirch, France).

Activation of DDR2 on 2D collagen coating: The effects of the aged type I collagen 2D coating on DDR2 activation were studied using 150 mm diameter culture dishes. Each culture dish was coated with 5 µg/cm² of adult or old collagen (as described in materials and methods section). Then, coated substrates were dried overnight at room temperature under sterile conditions and rinsed once in PBS (Invitrogen) before cell plating. HT-1080 cells were serum starved overnight, then seeded on plastic or the coated surfaces at a concentration of 1×10^7

cells/culture dish without FBS. After 6 hours, cells were lysed using RIPA buffer, and protein extracts were submitted to immunoprecipitation and western blotting experiments.

siRNA transfections: siRNA oligonucleotides were transfected in HT-1080 cells with Lipofectamine RNAiMax (Invitrogen) according to the manufacturer's instructions. A pool of 3 target-specific 19-25 nucleotides SHP-2 siRNAs (sc-36488) was purchased from Santa Cruz Biotechnology, and were used at 50 nM. Negative control siRNA (1027310) was purchased from Qiagen (Courtaboeuf, France). Cells were allowed to grow 24 hours after transfection without FBS before use. Then cells were seeded in type I collagen 3D matrices as described in materials and methods section. After 6 hours, cells were lysed RIPA buffer and SHP-2 extinction was controlled by western blotting.

References

1. Hammerman PS, Sos ML, Ramos AH, Xu C, Dutt A, Zhou W, Brace LE, Woods BA, Lin W, Zhang J, Deng X, Lim SM, Heynck S, Peifer M, Simard JR, Lawrence MS, et al. Mutations in the DDR2 kinase gene identify a novel therapeutic target in squamous cell lung cancer. Cancer Discov. 2011; 1(1):78-89.
2. Beauchamp EM, Woods BA, Dulak AM, Tan L, Xu C, Gray NS, Bass AJ, Wong KK, Meyerson M and Hammerman PS. Acquired resistance to dasatinib in lung cancer cell lines conferred by DDR2 gatekeeper mutation and NF1 loss. Mol Cancer Ther. 2014; 13(2):475-482.

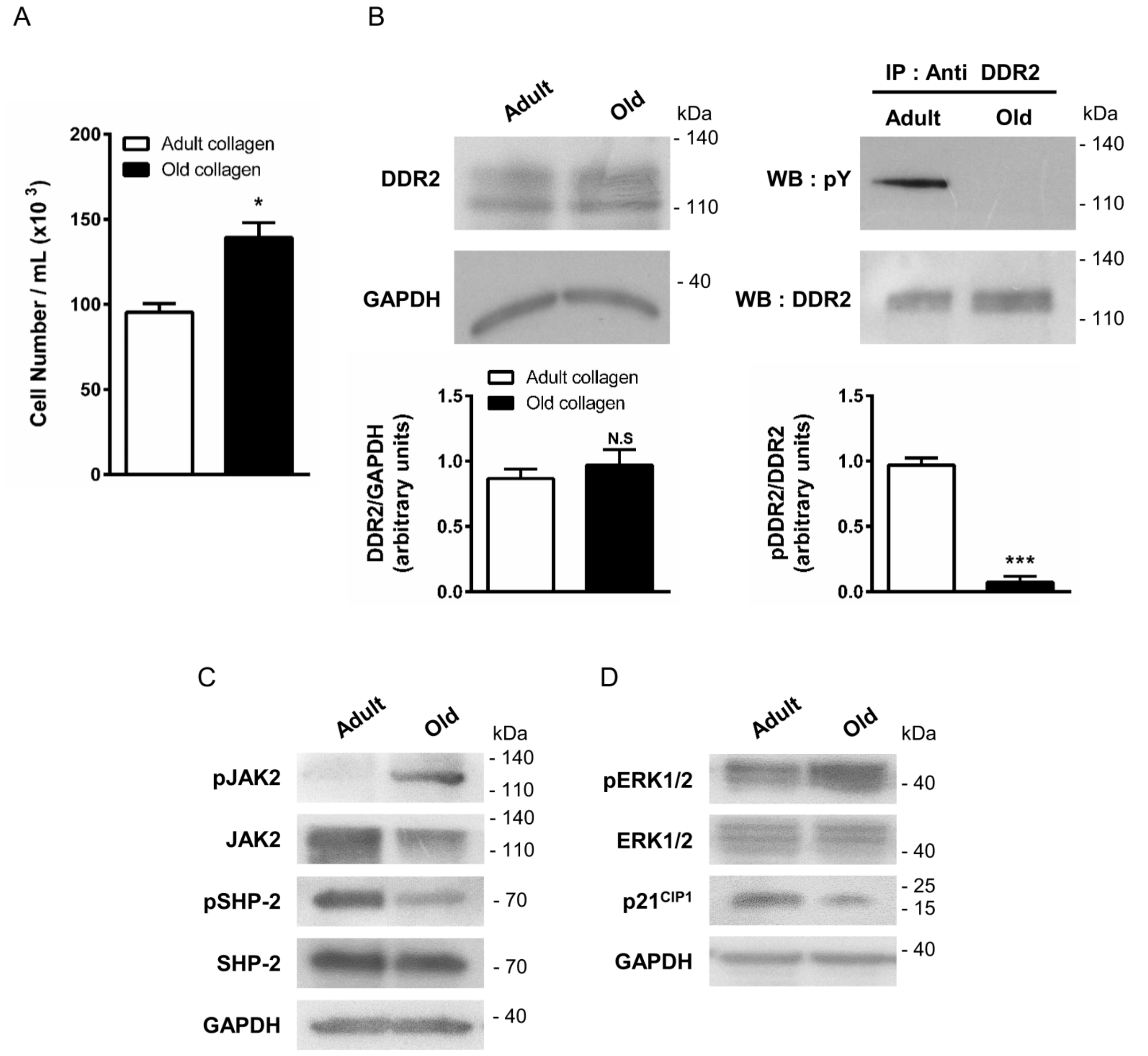

Supplementary data 1: Effect of collagen aging on A204 cell proliferation and signaling pathways in adult and old type I collagen 3D matrices.

(A) Cell proliferation in 3D matrices. Cells were seeded at a density of 1.5×10^4 cells/ml and cell number was evaluated after 5 days of culture by phase contrast microscopy. (B) DDR2 expression and activation. Cells were cultured 6 hours in 3D matrices without FBS and western blot analysis was performed using anti-DDR2 specific antibody (left panel). The histogram shows the ratio of DDR2 expression relative to the loading control GAPDH. Immunoprecipitation was performed using anti-DDR2 specific antibody, and DDR2 activation was measured by western blot using anti-phosphotyrosine specific antibody (right

panel). The histogram shows the ratio of pDDR2 expression relative to DDR2. (C) Expression and activation of SHP-2 and JAK2. Cells were seeded in 3D matrices and western blot analysis was performed after 6 hours and 5 days for SHP-2 and JAK2 respectively using specific antibodies. (D) Activation and expression of ERK1/2 and $p21^{CIP1}$. Cells were seeded in 3D matrices and western blot analysis was performed after 5 days using specific antibodies. Data represent the mean ± S.E.M. of three independent experiments (N.S. = not significant, *$p<0.05$, ***$p<0.001$).

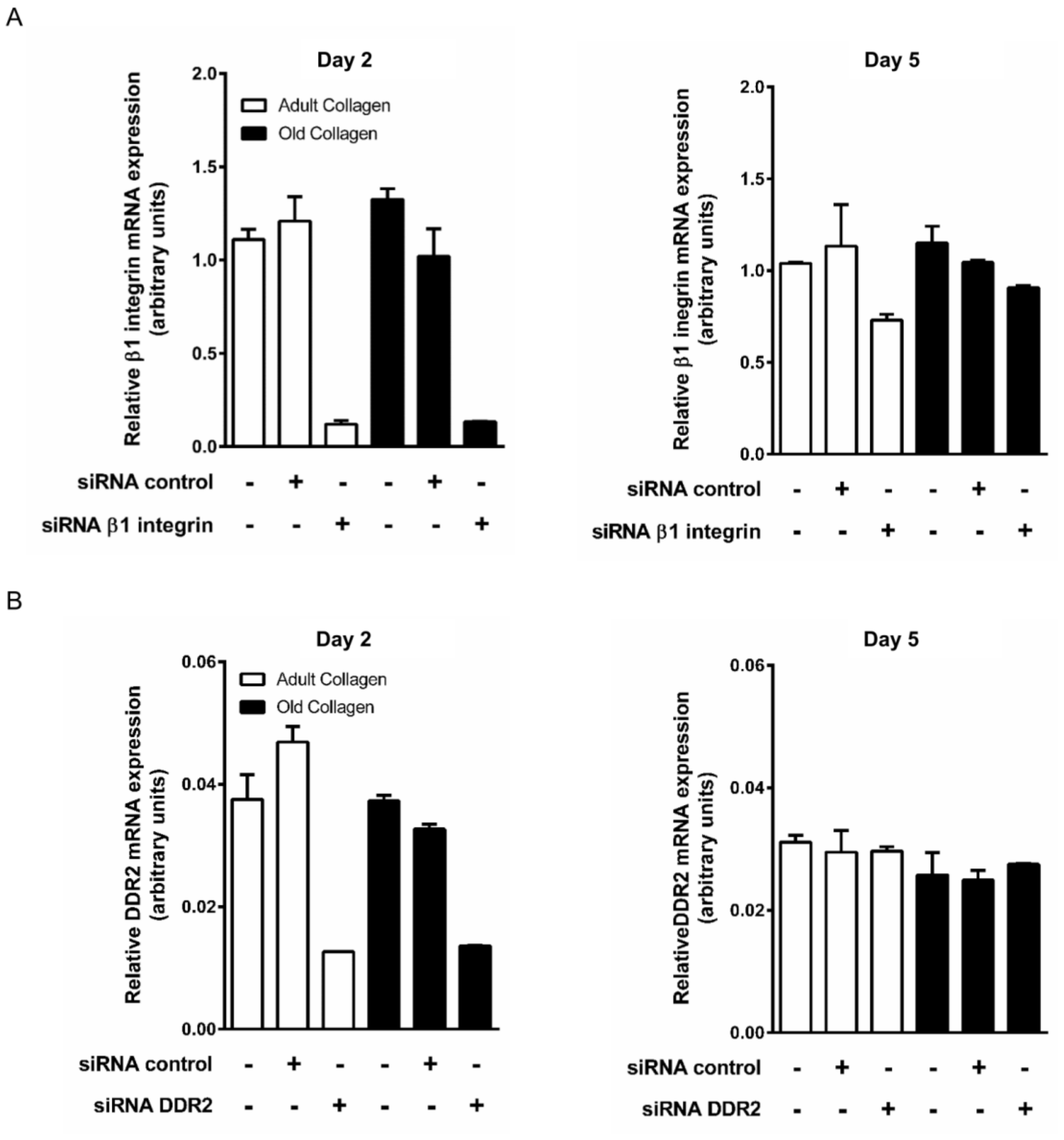

Supplementary data 2: β1 integrin and DDR2 expression in HT-1080 after 2 and 5 days of siRNA treatment.

HT-1080 cells were treated with siRNA against β1 integrin or DDR2, and then were cultured in adult and old collagen 3D matrices up to 5 days. (A) β1 integrin transcripts content in HT-1080 cells was evaluated by real-time quantitative polymerase chain reaction after 2 days (left panel) and 5 days (right panel). (B) DDR2 transcripts content in HT-1080 cells was evaluated by real-time quantitative polymerase chain reaction after 2 days (left panel) and 5 days (right panel). Data are representative of three independent experiments.

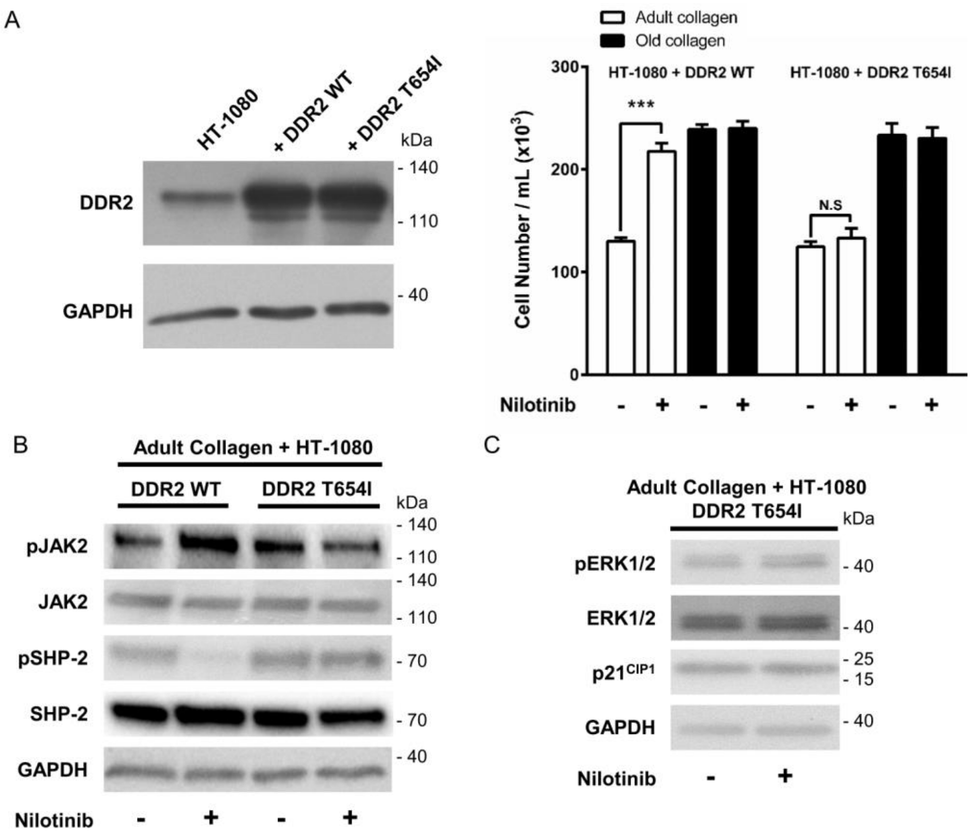

Supplementary data 3: Effect of collagen aging on cell proliferation and signaling pathways in wild-type and gatekeeper DDR2 transfected HT-1080 cells in 3D matrices.

(A) DDR2 expression (left panel) and cell proliferation in the presence or not of nilotinib (right panel). DDR2 protein expression was analyzed by western blot and GAPDH was used as a loading control. For cell proliferation, cells were seeded in 3D matrices at a density of 1.5×10^4 cells/ml. After 5 days of culture in the presence or not of nilotinib, cell density was evaluated by phase contrast microscopy. (B) Expression and activation of SHP-2 and JAK2. Cells were seeded in 3D matrices in the presence or not of nilotinib and western blot analysis was performed after 6 hours and 5 days for SHP-2 and JAK2 respectively using specific antibodies. (C) Activation and expression of ERK1/2 and p21^{CIP1}. Cells were seeded in 3D matrices in the presence or not of nilotinib and western blot analysis was performed after 5 days using specific antibodies. Data represent the mean ± S.E.M. of three independent experiments (***p<0.001, N.S. = not significant).

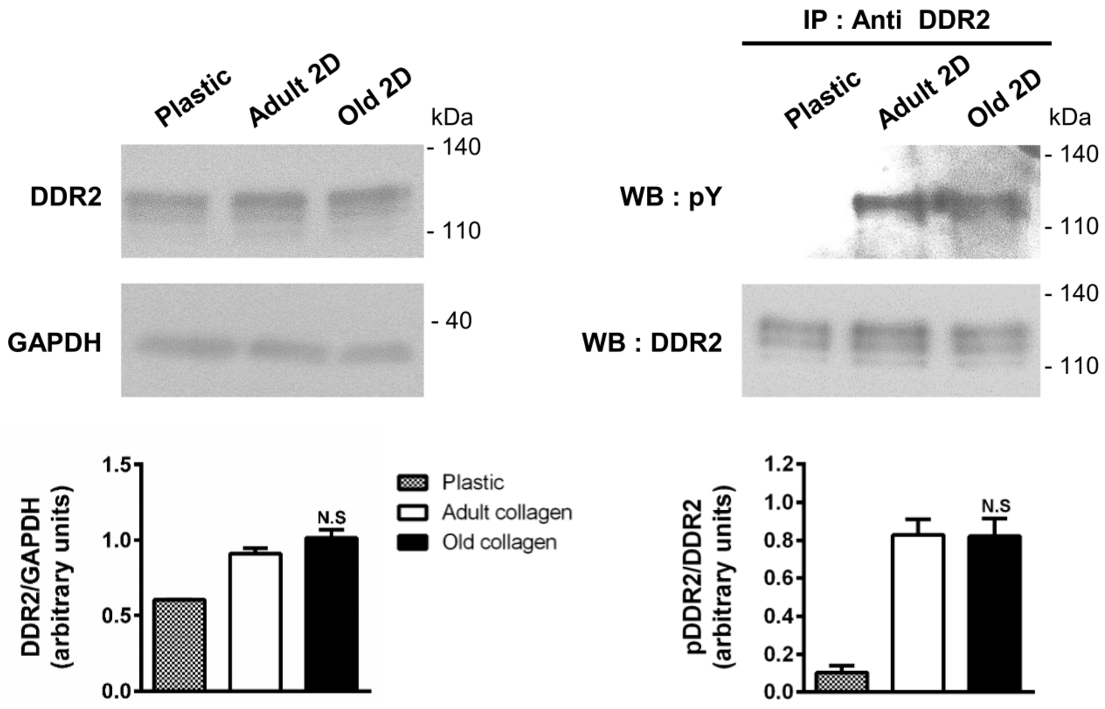

Supplementary data 4: Effect of collagen aging on DDR2 expression and activation on 2D coating.

DDR2 expression (left panel) and activation (right panel) on collagen 2D coating. Cells were cultured 6 hours on plastic or collagen 2D coating without FBS and western blot analysis was performed using anti-DDR2 specific antibody (left panel). The histogram shows the ratio of DDR2 expression relative to the loading control GAPDH. Immunoprecipitation was performed using anti-DDR2 specific antibody, and DDR2 activation was measured by western blot using anti-phosphotyrosine specific antibody (right panel). The histogram shows the ratio of pDDR2 relative to DDR2. Values represent the mean ± S.E.M. of three independent experiments (N.S. = not significant).

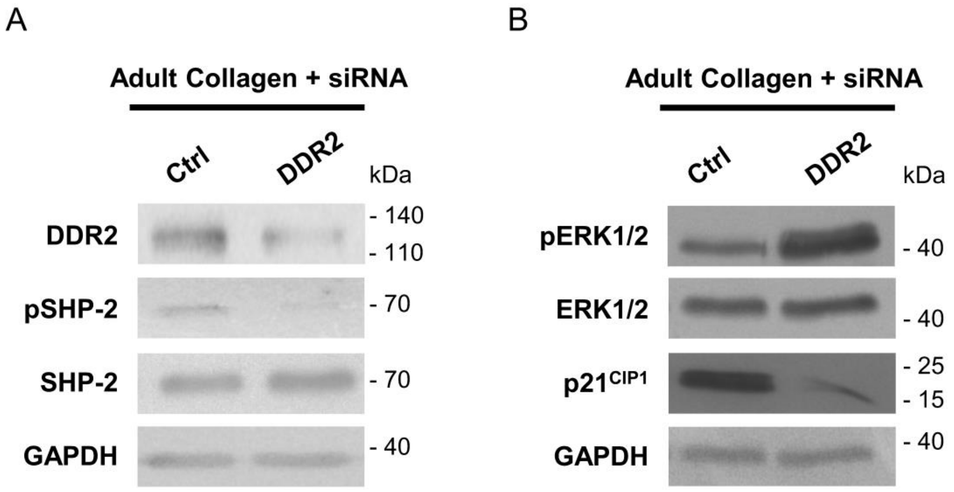

Supplementary data 5: Effect of DDR2 depletion on signaling pathways in adult collagen 3D matrices.

(A) Expression and activation of SHP-2. Cells were seeded in adult collagen 3D matrix without FBS for 6 hours after DDR2 depletion by siRNA strategy, and western blot analysis was performed using specific antibodies. (B) Activation and expression of ERK1/2 and $p21^{CIP1}$. Cells were seeded in adult collagen 3D matrix for 5 days after DDR2 depletion by siRNA strategy and western blot analysis was performed using specific antibodies. Data are representative of three independent experiments.

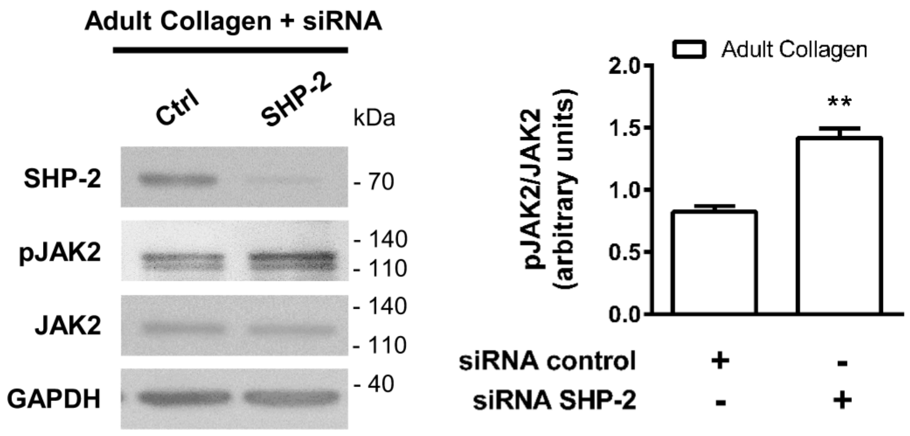

Supplementary data 6: Effect of SHP-2 depletion on JAK2 phosphorylation in adult collagen 3D matrices.

Cells were seeded in adult collagen 3D matrix without FBS for 6 hours after SHP-2 depletion by siRNA strategy, and western blot analysis was performed using specific antibodies. The histogram shows the ratio of pJAK2 relative to JAK2. Values represent the mean ± S.E.M. of three independent experiments (**p<0.01).
